# Conservative management of post-appendicectomy intra-abdominal abscesses

**DOI:** 10.1186/1824-7288-36-68

**Published:** 2010-10-14

**Authors:** Mahdi Ben Dhaou, Sofiene Ghorbel, Taieb Chouikh, Awatef Charieg, Faouzi Nouira, Sonia Ben Khalifa, Rachid Khemakhem, Said Jlidi, Béji Chaouachi

**Affiliations:** 1 Department of paediatric surgery B, Children Hospital of Tunis, 1007 Tunis. Tunisia; 2 Department of anaesthesia reanimation. Children Hospital of Tunis, 1007 Tunis. Tunisia

## Abstract

**Purpose:**

Appendicitis is the most common abdominal inflammatory process in children which were sometimes followed by complications including intra-abdominal abscess. This later needs classically a surgical drainage. We evaluated the efficacy of antibiotic treatment and surgical drainage.

**Methods:**

Hospital records of children treated in our unit for intra-abdominal post appendectomy abscesses over a 6 years period were reviewed retrospectively.

**Results:**

This study investigates a series of 14 children from 2 to 13 years of age with one or many abscesses after appendectomy, treated between 2002 and 2007. Seven underwent surgery and the others were treated with triple antibiotherapy. The two groups were comparable.

For the 7 patients who receive medical treatment alone, it was considered efficient in 6 cases (85%) with clinical, biological and radiological recovery of the abscess. There was one failure (14%). The duration of hospitalization from the day of diagnosis of intra-abdominal abscess was approximately 10.28 days (range 7 to 14 days). In the other group, the efficacy of treatment was considered satisfactory in all cases. The duration of hospitalization was about 13 days (range: 9 to 20).

**Conclusion:**

Compared to surgical drainage, antibiotic management of intra-abdominal abscesses was a no invasive treatment with shorter hospitalization.

## Introduction

Appendectomy is considered to be a surgical intervention with a low morbidity and mortality rate [1]. However, sepses remain common post-appendectomy complication which represent 2% of cases post-operatively [2-4]. The occurrence of intra-abdominal abscesses is considered as the most serious one, for which the traditional treatment is surgical drainage [5]. We hypothesized that most of these abscesses can be successfully managed by antibiotic treatment alone. The aim of this study is to evaluate the efficacy of antibiotic management compared to classic surgical treatment.

## Patients and Methods

A retrospective review of 14 children who developed intraabdominal abscess after initial appendicectomy, following 1400 appendectomy practiced in our Department between 2002 and 2007. Children presenting with a post-appendectomy intra-abdominal abscess who were not originally operated in our unit were excluded.

We reviewed the hospital records of these children in order to collect the following information: age and sex, operative finding of the original appendectomy, clinical symptoms, blood parameters indicating infection, ultrasonographic signs such as location and size of the abscesses, definitive treatment of the abscess and the follow up.

All of these patients were operated by open surgery either for the first surgery (for the two groups) or the second ones when those patients were reoperated.

All patients received systematic intravenous antibiotic treatment. In cases with no complicated appendicitis, amoxicillin-clavulanic acid (50-100 mg/Kg/d), Gentamicin (3-5 mg/Kg/d) were administered only during the intervention. Complicated appendicitis (gangrenous, abscesses or peritonitis) was treated postoperatively with the same antibiotics for 5 to 10 days depending on the clinical evolution of the patient.

An intra-abdominal postoperative abscess was suspected in cases with increasing abdominal pain, intestinal obstruction, diarrhea and persisting, increasing pyrexia developing after appendectomy, or after biological signs, like high level of leukocytes and augmentation of C-reactive protein. When the diagnosis of abscess was suspected, the patient had systematically an abdominal ultrasonography which confirmed diagnosis. Then, we started the antibiotic treatment with Cefotaxim (100 mg/kg/d), Gentamicine (3-5 mg/kg/d) and Metronidazole (20 -30 mg/kg/d). For evaluating the result of treatment, we took the following into account: the evolution of initial clinical signs, ultra sonographic reassessment of the size of the abscess and the evolution of biological parameters. These two later procedures were evaluated every four days. Antibiotic treatment was stopped when the clinical examination did not reveal symptoms of the abscess: 48 hours without fever, the normality of CRP and leukocytes and radiological recovery. The patients were discharged when they no longer presented any clinical or laboratory signs. Follow-up consisted of repeated ultrasound and clinical examination at 15 and 90 days after discharge.

## Results

There were 7 girls and 7 boys (Table 1, 2), aged from 2 to 13 years (mean: 8 years). All of these patients had no particular medical or chirurgical antecedents. Appendicular abscess was found in four cases and peritonitis in eight and no complicated appendicitis in 2 cases. The infection complication was diagnosed between the third and the twelfth post operative day (mean: 6 days). Clinical signs present at diagnosis were the following: fever in 13 cases (92%), abdominal pain in 13 (92%), transit problems (vomiting, diarrhea, blockage) in 11 cases (78%), and isolated biological modification in one case (7%). Diagnosis confirmation, localization and measure of the abscess were performed by ultrasonography in 13 cases and CT scan in one. Solitary abscesses were located in Douglas' pouch in 5 cases, in the right iliac fossa in 2 cases, subphrenic area in 2 cases and right parietocolic gutter in 3 cases. Two patients presented with multiple abscesses, measured between 17 mm and 90 mm at their widest point.

**Table 1:** Characteristics of the 2 groups

	Antibiotics	Surgery+antibiotics
Number of patients	7 (50%)	7 (50%)

Mean age (year)	8,7	8,7

Mean duration of hospitalization (days)	10,28	13

**Mean size of abscess (mm)**	39,85	46,11

**Table 2:** Localizations of the abscesses

Localization of abscess	Surgical treatment	Medical treatment
Douglas' pouch	4	1
Right iliac fossa	1	1
Sub phrenic area		2
Right parieto-colic gutter	2	1
Multiple		2

The initial treatment was surgical and medical in 7 cases and only medical in the 7 others.

In the 7 patients receiving medical treatment alone, apyrexia was obtained within 3 days after the start of treatment. The clinical signs disappeared within 5 days following begin of treatment; diet was started 4 days after instauration of treatment. The efficacy of treatment was considered good in 6 cases (85%) with clinical, biological and radiological recovery of the abscess. There was one failure, where an abscess relapsed despite several courses of antibiotherapy. It was a 13-year-old boy who was admitted for acute appendicitis, operative exploration showed appendicular abscess. Five days later, because of the remaining fever and biological signs (leukocytes of 16000/mm3 and a CRP count 60 mg/l), ultrasonography examination was practiced and showed multiple abscesses with one major abscess (6,5 × 8 cm). The patient received antibiotic treatment for 10 days. Unfortunately, no clinical or radiological amelioration was observed. Releasing of appendicular base was suspected and surgical intervention was practiced with uneventful recovery. The patient was discharged 18 days after second surgery.

The duration of hospitalization from the day of diagnosis of intra-abdominal abscess was approximately 10.28 days (range 7 to 14 days)

All the children who received medical treatment were discharged 24 hours after the end of antibiotic treatment. We did not observe any complications in these children. None of the children required a surgical intervention. A relapse was not observed in the follow-up examination in any cases.

Seven of the 14 children with post-appendectomy intra-abdominal abscesses were treated either by drainage (1 patient) or surgical intervention. In those cases, fever disappeared on average within 3 days. The clinical signs disappeared within the 3 days following the beginning of treatment. The efficacy of treatment was considered satisfactory in all cases with clinical, biological and radiological recovery of the abscess. The duration of hospitalization from the day of diagnosis of intra-abdominal abscess was 13 days (range: 9 to 20).

In one case, scan-guided drainage was carried out. This was in a 3-year-old girl where operative exploration showed appendicular abscess, because of fever persisting with a leukocytes of 15600/mm3 and a CRP count 170 mg/l, abdominal ultrasound was practiced and showed a sub-phrenic abscess (43 × 21 mm) which was immediately drained. The patient received antibiotic treatment for 7 days without amelioration. Surgical intervention was then indicated and the patient was discharged at day 18 post operative.

## Discussion

Recurrent abscesses after initial or delayed appendicectomy for perforated appendicitis with abscesses occurs in about 17% of the cases [6].

Choosing probabilistic antibiotherapy implied having good knowledge of the bacterial profile of deep abscesses. The choice of an appropriate antibiotic is a major issue. In peritoneal infections secondary to appendicitis, several germs may often occur, so probabilistic antibiotherapy should follow several rules, triple antibiotherapy is recommended in order to avoid any bacterial resistance and broaden the spectrum of action [7]. It should combine antibiotics which are active against the aforementioned germs. Bactericide antibiotics may also be used due to their rapid action and the synergy of their combination. One of the antibiotics used must be effective against anaerobic germs. Tissue diffusion must be good and the route of administration must be intravenous, at least in the beginning, because of the frequent risk of digestive intolerance. Finally, any antibiotic used in children must have no important side effects. Therefore, we chose the following protocol: third-generation cephalosporin + aminoside + imidazole.

In our study, the chosen triple therapy was efficient in 86% of children who received only conservative treatment. No side effects or complications were observed.

The result of this study support studies where conservative treatment of intra- abdominal abscesses following appendectomy is also recommended [3,7-11]. Gorenstein and al. reported complete resolution of a post-appendectomy intra-abdominal abscess in 8 of 10 patients with only the use of antibiotics [9].

The cure of 10 of 11 children with intra-abdominal abscess following appendectomy using exclusively medical treatment was demonstrated in the study of Heloury and al [10]. In our study, antibiotherapy was successful in 6 of 7 children.

The last study dealing with this subject compared the evolution of 11 children presenting with an intra-abdominal abscess treated by surgical intervention with a comparable group of 11 children treated for intra-abdominal abscess with antibiotics alone [8]. This study found a shorter hospital stay (10.4 days versus 16.7 days) as well as a lower complication rate in the children treated conservatively.

The classical approach is to drain any deep intra-abdominal abscess occurring post operatively [2-11] by surgery or percutaneous assisted drainage with computed tomography or ultrasound guidance. Drainage is an invasive procedure [3] and requires re-intervention through a larger route in a child who is already tired. It was the cases of 3-year-old boy which show that the drainage tends to be relatively dangerous, a point of view confirmed by Heloury [10] who reported between 25% to 45% complication rate occurring after drainage of deep abscesses in a series of patients aged from 4 to 84 years according to their localization.

The result of this study support the hypothesis that most post appendectomy intra-abdominal abscesses can be successfully managed by intravenous antibiotic therapy alone.

## Conclusion

Abscesses continue to remain an important clinical problem. The great advances in ultrasonography techniques help on the diagnosis and the follow-up. Appropriate antibiotic treatment can be recommended as an efficacious first line treatment in cases of post appendectomy intra-abdominal abscesses.

## Competing interests

The authors declare that they have no competing interests.

## Authors' contributions

MD, SG, TC and AC have contributed to the collection of specific features of the cases as well as operator in the surgery of these children they also have contributed to the conception of the study, and participated in its design and coordination.

FN, RK, SJ and BC contributed as surgeons

SB contributed as a chief of the department of anaesthesia reanimation to adapt the antibiotic therapy and regular monitoring of these patients

All authors read and approved the final manuscript
